# Rasch analysis of the PedsQL 3.0 diabetes module

**DOI:** 10.1186/1745-6215-14-S1-O40

**Published:** 2013-11-29

**Authors:** Tim Pickles, Rebecca Playle, Kerenza Hood, Jonathan Gillard, Michael Robling

**Affiliations:** 1 South East Wales Trials Unit, Cardiff University, Cardiff, UK; 2 Cardiff School of Mathematics, Cardiff University, Cardiff, UK

## 

Rasch analysis is an increasingly popular approach to questionnaire reduction and the validation of outcome measures, and is rapidly overtaking the standard classical test theory approaches of Cronbach's alpha and Factor Analysis. Data from outcome measures, in which the items are intended for summation to create an overall ordinal score, are tested against the expectations of the Rasch measurement model. Rasch analysis provides numerous fit statistics to show how well the various items describe the group of individuals, and how well those individuals fit the group. Further issues include person separation, targeting, threshold ordering, differential item functioning, local independence and unidimensionality, which can be explored and interpreted from the output generated by software package RUMM2030.

Rasch analysis was undertaken on data from the PedsQL 3.0 Diabetes Module (PQDM), which was completed by patients, and carers of patients, as part of two large randomised controlled trials of interventions in paediatric type 1 diabetes patients. Initial results suggest that the PQDM in its current format is not an adequate measure of diabetes-specific health-related quality of life, with all 5 scales showing some form of misfit to the Rasch measurement model. Attempts to improve fit will be presented, demonstrating how Rasch analysis can be utilised to amend the outcome measures. By reworking this measure, and indeed any other measure, via Rasch analysis, we will have increasingly more psychometrically relevant, numerically valid and statistically reliable measures to use as trial endpoints, making greater use of accrued trial data in secondary analyses.

